# Eufemiusz J. Herman: A Pioneer and Leader in Polish Neurology

**DOI:** 10.7759/cureus.68685

**Published:** 2024-09-05

**Authors:** Kacper Mazurkiewicz, Leon Pawlik, Martyna Musiorska

**Affiliations:** 1 Research, Central Clinical Hospital of the Medical University of Lodz, Lodz, POL

**Keywords:** eufemiusz herman, historical vignette, history of neurology, medical scientist, neurology, polish neurologist

## Abstract

Eufemiusz J. Herman was a pioneering figure in Polish neurology whose contributions spanned clinical practice, research, and education. Born in 1892, his career was marked by a deep understanding of neurological semiology, which he honed under the mentorship of Edward Flatau. Herman was a true scientist and physician, demonstrating his dedication to research even before graduating from medical school. His commitment to scientific inquiry persisted even during the harrowing conditions of the Warsaw Ghetto, where, amidst an epidemic of typhus, he documented and treated the neurological complications of the disease. His extensive body of work, comprising 17 books and over 200 scientific papers, led to the description of several enduring clinical signs, including the eponymous Herman's syndrome (post-traumatic syndrome with livedo racemosa universalis) and the nuchal-toe sign in meningitis. Herman's enduring legacy encompasses not only his scientific discoveries but also his pivotal role in shaping Polish neurology. His work bridged pre- and postwar neurological traditions, laying the foundation for modern neurological practice in Poland and contributing to the international advancement of the field. This paper reviews Herman's most noteworthy scientific achievements and their impact on neurological practice.

## Introduction and background

Early life and medical education

**Figure 1:**
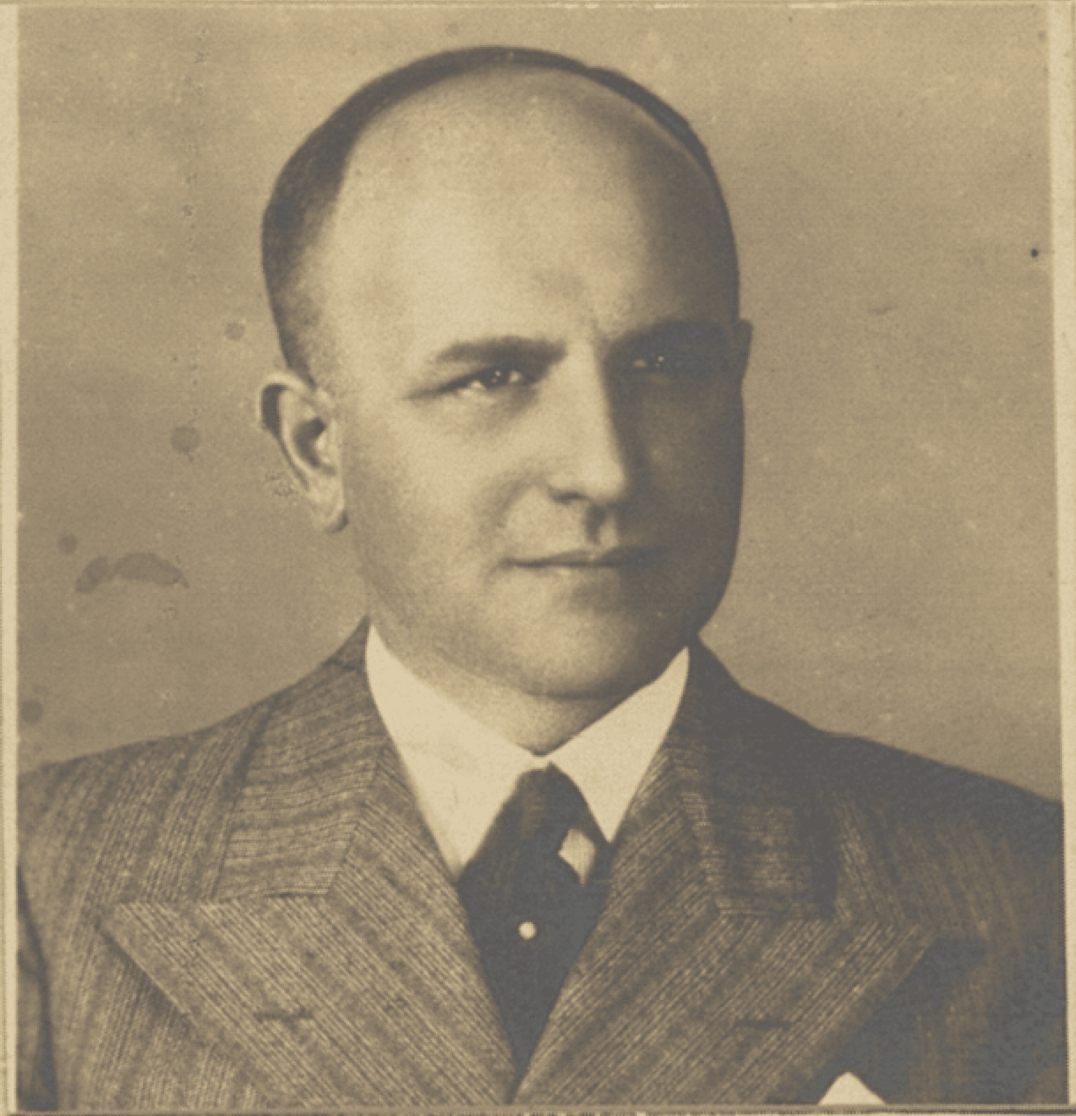
Portrait of Eufemiusz J. Herman Image credit: Permisson obtained from the Main Medical Library, Warsaw. Signature: DSKM GBL PL\327\1\0\1871

Eufemiusz Józef Herman (Figure 1) was born on September 29, 1892, in Tomaszów Mazowiecki, a town then under Russian occupation in Poland. His parents, Jakub and Helena Herman, were both of Jewish heritage. They were primary school teachers, which likely instilled in him a strong intellectual foundation from an early age. He pursued medical studies at John Casimir University in Lviv and Jagiellonian University in Cracow. During the First World War, as a third-year medical student, he briefly left Lviv for Lodz where he volunteered at the “Kochanówka” Hospital under Antoni F. Mikulski [1]. He later resumed his studies as a fourth-year medical student in Cracow, where he published some of his earliest scientific papers under the mentorship of Jan Pilz [2]. Notably, even before earning his degree, he had already published several papers, including studies on peripheral nerve endings in mammals, proteinuria in brain tumors, cerebellar tumors, and brain pulsation [3]. He received his medical diploma in November 1918. After graduation, he continued to release publications on schizophrenia, proprioception, and other subjects [1].

Soviet-Polish war

In 1920, during the Polish-Soviet war, he joined the Polish Army as an officer. He served in the Epidemical Hospital of the Polish Army No 2 in Brest-on-the-Bug where he managed an outbreak of epidemic typhus. As the war drew to a close, he was stationed at Mokotow Hospital in Warsaw [4]. After the war, Herman was demobilized and chose to settle in Warsaw, where he began working in the Neurological Department of The Jewish Hospital in Czyste. This marked the beginning of a significant chapter in his career.

## Review

Early career and mentorship at the Jewish Hospital

In 1922, Eufemiusz J. Herman began working under the mentorship of the renowned physician and scientist Edward Flatau, a pivotal moment that would shape the trajectory of his career. He spent two decades, from 1922 to 1942, at the Neurological Department of the Jewish Hospital in Czyste. Following the death of Ludwik Bergman in 1932, Herman took over as the head of the second neurological department. During that time, he published around 50 scientific papers on topics such as intracranial calcifications and neuralgias, contributing to Polish, German, and French journals. Under Flatau's guidance, he developed a profound understanding of neurological semiology, mastering the ability to correlate observed symptoms and reflexes with specific locations of damage in the central nervous system. Apart from his inpatient and outpatient work, he worked at the Nencki Institute in Warsaw headed by Kazimierz Orzechowski [3].

World War II and the Warsaw Ghetto

During World War II, Eufemiusz J. Herman was once again mobilized to the Polish Army and incorporated into 9. Regional Hospital in Brest-on-the-Bug. He served until the autumn of 1939. After the end of the September Campaign, he returned to German-occupied Warsaw. Due to his Jewish heritage, he and his family were forced to live in the ghetto [5]. There he showed his high morals and irresistibility by not withholding from his work and even maintaining his scientific interests. Here, he again was fighting epidemic typhus that was taking an enormous toll in the ghetto. The Jewish Hospital, which was located within the ghetto walls, was at that time dedicated exclusively to treating typhus patients. The neurological department, led by Herman, was primarily devoted to those with severe neurological complications. During this outbreak, he contracted the disease himself. Amidst the harrowing realities of wartime, he gathered data regarding typhoid neurological complications which culminated in a scientific publication in 1949, following the war [6]. In 1942, he managed to escape the ghetto and remained hidden until the end of German occupation.

Postwar period in Łódź

After the war, in 1945, he settled in Łódź, one of few cities relatively untouched by the war. He was appointed director of the “Kochanówka” Psychiatric Hospital (where he volunteered as a medical student many years ago). On February 14, 1946, he was appointed head of the Neurological Department at the newly established Faculty of Medicine at the University of Łódź by the President of the State National Council, thus becoming an associate professor. To ensure that his title of associate professor was not seen as a result of circumstantial factors, Professor Herman undertook and completed his habilitation process in 1946 at the Faculty of Medicine of the Warsaw University [3].

In 1947, to provide multidisciplinary care for patients (Kochanówka was a hospital specializing only in neurological and psychiatric care), he decided to relocate the Neurological Department to Social Insurance Hospital (now Norbert Barlicki University Teaching Hospital No. 1 in Łódź) [7].

Subsequently, he was awarded two scholarships: one from the Svenska Institute, which allowed him to spend two months practicing at Herbert Olivecrona’s Neurosurgical Clinic in Stockholm, and another from Columbia University, enabling him to spend six months at the Neurological Clinic in New York. During his time across the Atlantic, he presented his findings on typhus studies conducted in the Warsaw Ghetto [5]. Upon returning to his homeland, due to new regulations from the Ministry of Education, which required the completion of a doctoral degree before obtaining the title of professor, Herman initiated a doctoral process, which he successfully defended in 1955 (despite already holding the titles of habilitated doctor and associate professor). The following year, in 1956, the Senate of the Medical Academy of Łódź awarded him a professor title.

**Figure 2:**
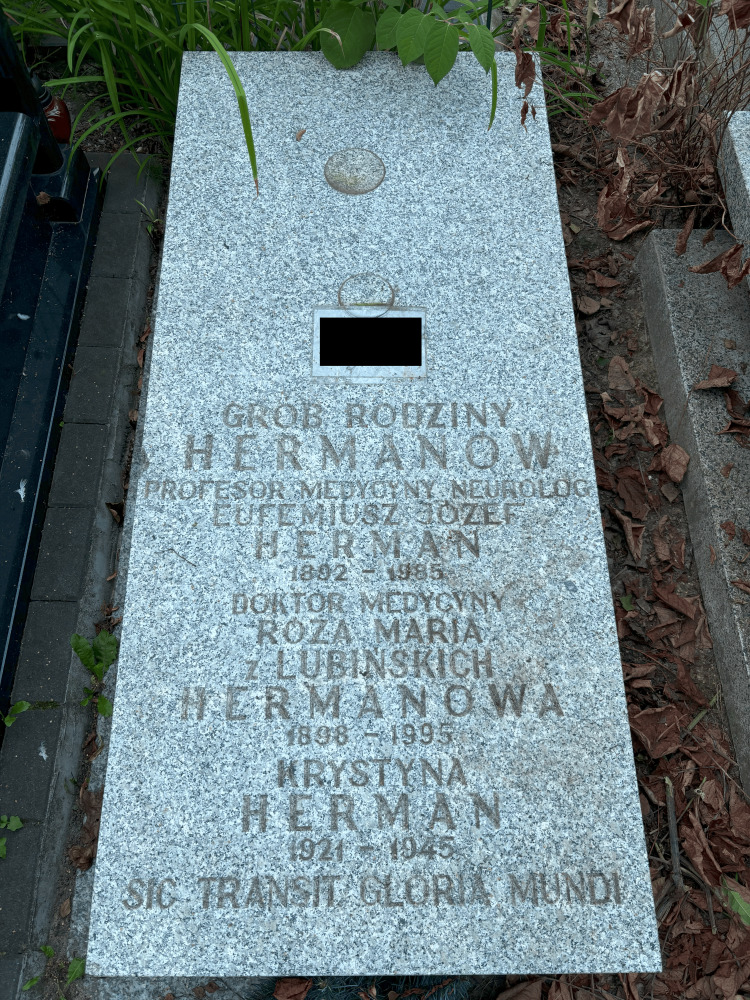
Eufemiusz Herman's grave at the old cemetery in Łódź The Herman family grave at the old cemetery in Łódź, where Eufemiusz J. Herman, his wife Róża Maria Herman, and their daughter Krystyna Herman are buried. Image credits: Kacper Mazurkiewicz

Despite retiring in 1962, Herman remained actively engaged with the department, arriving at his office each morning at eight o’clock [3]. In 1975, he was honored with an honorary doctorate from the Medical Academy of Łódź. He passed away on May 8, 1985. He was married to Roza Maria Lubinska, who was also a physician and an exceptional chess player. Their only daughter, Krystyna Herman, was reportedly stoned to death by her boyfriend and his colleagues in 1945 after she revealed her Jewish heritage, which she had been concealing under a false identity [8]. The Herman family’s grave is located on Ogrodowa Street in the Old Cemetery of Łódź (Figure 2).

Legacy and contributions to neurology

**Figure 3:**
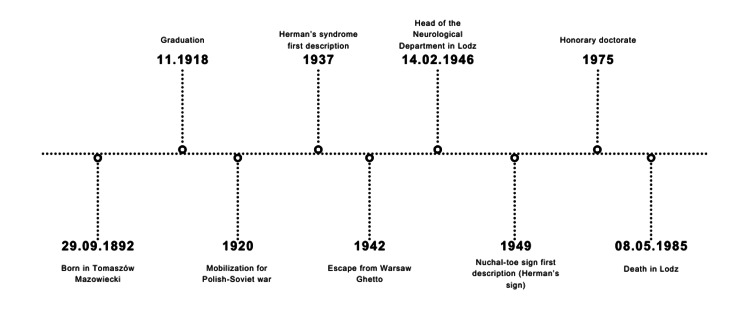
Chronology of Eufemiusz J. Herman's life Timeline highlighting key events in the life of Eufemiusz J. Herman. Image credits: Kacper Mazurkiewicz

Eufemiusz J. Herman was a prolific author, having written 17 books and more than 200 scientific papers. His exceptional observational skills and clinical reasoning, combined with his lifelong curiosity and dedication to research, led him to be the first to describe numerous symptoms and signs that became invaluable for bedside examination (Figure 3).

His pupils described him as a neurologist of a bygone era, one who possessed a profound understanding of anatomy and neurological semiology. He belonged to a generation that brought the clinical examination to its zenith at the dawn of an era increasingly dominated by imaging studies.

Among most important clinical signs described by Herman are the follow-up sign in hemiparesis (work for which he received his habilitation degree) [1], nuchal-toe sign in meningitis (known as Herman’s sign) [9], tonic plantar reflex (typical for frontal lobe tumors) [10], neurological syndromes in typhus [6], the intermittent reflexes in myasthenia [3] (diminishing deep tendon reflexes with repeated stimulation, a diagnostic clue that can be detected during routine clinical examination), post-traumatic syndrome with livedo racemosa universalis (so-called Herman’s syndrome) [11], the ischemic test in myasthenia (a maneuver that increases the diagnostic yield of electromyography) [12], and the sign of unilateral upper lid retraction in internuclear ophthalmoplegia [13]. 

Notably, two of these signs are eponymously named and represent Herman's most enduring clinical contributions. Herman’s sign, although relatively rare, is a useful indicator of meningismus, particularly noted in tuberculosis meningitis with involvement of both leptomeninges and cortex [9, 14]. Herman’s syndrome is a neurological post-traumatic syndrome characterized by universal livedo racemosa, pyramidal and extrapyramidal signs, speech disturbances, mental disorders, and arterial hypertension [11]. This syndrome, first described in 1937, then discussed by Herman and Sulat in 1957 and 1959, shares some similarities with the more widely recognized Sneddon syndrome, which was not described until 1965 [15].

In his memories of working at the Norbert Barlicki University Teaching Hospital Herman recalls presenting his discovery of a new sign of meningitis at the Fourth International Neurological Congress. During the event, Professor Robert Wartenberg reportedly expressed his admiration, stating he was pleased that a Pole had made this discovery. Wartenberg also added, "I do not speak Polish, but I know this one phrase: “Poland has not yet succumbed!”"-a line taken from the Polish national anthem [16].

He also authored several major publications, including a detailed volume on inflammatory diseases of the brain [17], an extensive description of neurological syndromes in rheumatological diseases [18] and a chapter on congenital, early-acquired, and familial nervous system disorders [19].

His handbook on the diagnosis of neurological diseases stands as a testament to his excellence in neurological semiology. It was reprinted several times and is still remembered among subsequent generations of Polish neurologists. True to his slogan, “You are a physician first, and only then a specialist” [3], he also published a handbook on neurological syndromes in internal medicine [20], which was translated into German and Russian languages [21,22].

His works on the history of neurology are held in great esteem and serve as crucial sources of knowledge that might otherwise have been lost. Notably, he wrote a thorough biography of worldwide known neurologist of Polish descent Joseph Babiński (1965) [23] and two seminal books on the history of Polish neurology (1975) [24] and Polish neurologists (1958) [25]. In these works, he profiled over a hundred exceptional neurologists, including figures he personally knew, such as Eduard Flatau, Samuel Goldflam, Joseph Babiński, and Jan Piltz. He also participated in the first International Neurological Congress held in Bern, where he is featured in a surviving recording (Video 1).

**Video 1:** Eufemiusz J. Herman at the first International Neurological Congress held in Bern, 1931 Eufemiusz J. Herman is visible as the fourth figure from the left. Source: Courtesy of the Galter Health Sciences Library & Learning Center, Northwestern University Feinberg School of Medicine, Chicago, IL, USA [26]. Permission was obtained to share and shorten the original video. Credit: Photographed by S. W. Ranson for the Institute of Neurology, Northwestern University Medical School

Apart from his research and clinical contributions, he played a pivotal role in establishing an important new medical faculty in postwar Poland in Łódź. He served as Deputy Dean at the Medical Academy of Łódź from 1952 to 1954 [27]. He was dedicated to engaging the younger generation of physicians in scientific pursuits, actively participating in the work of the Student Scientific Society at the Medical Academy of Łódź [28]. He also established a Scientific Research Center for the treatment of neuroses in Kudowa-Zdrój [7].

He remained active in the national and international neurological community. In 1957, he was elected vice president of the International Neurological Congress in Brussels. He was elected president of the Polish Neurological Society in 1961 and 1966 [29]. In the years 1969-1972, he was vice president of the World Federation of Neurology. Paying testament to his interest in the history of neurology, he remained active within the Commission on the History of Neurology of the World Federation of Neurology. Additionally, he was an honorary member of several international societies, including the French Society of Neurologists (1956), Italian Society of Neurology (1956), and the Royal Society of Medicine (1957) [3]. Herman maintained connections with world-renowned neurologists. Under his leadership, the Department hosted many prominent international scholars, including several presidents of the World Federation of Neurology: Professor van Bogaert, Dr. Critchley, and Professor Refsum [7]. In 1967, to celebrate the 50th anniversary of Professor Herman's medical career, his colleagues and students commissioned a commemorative volume titled Neurological Problems, edited by Professor J. Choróbski [30].

## Conclusions

Eufemiusz J. Herman was a pioneering figure in Polish neurology. His keen clinical observations led to the identification of several eponymous signs, significantly advancing neurological diagnosis. As an educator and author, he shaped postwar neurology in Poland. His resilience during World War II underscores the importance of preserving medical knowledge in challenging times. Herman's legacy continues to influence contemporary neurology.
